# Tunicamycin Induces Hepatic Stellate Cell Apoptosis Through Calpain-2/Ca^2 +^-Dependent Endoplasmic Reticulum Stress Pathway

**DOI:** 10.3389/fcell.2021.684857

**Published:** 2021-09-17

**Authors:** Haiying Liu, Linyu Dai, Ming Wang, Fumin Feng, Yonghong Xiao

**Affiliations:** Department of Epidemiology and Health Statistics, School of Public Health, North China University of Science and Technology, Tangshan, China

**Keywords:** tunicamycin, calpain-2, apoptosis, endoplasmic reticulum stress, hepatic stellate cells

## Abstract

It has been reported that calpain/caspase-mediated apoptosis induced by endoplasmic reticulum stress (ERS) in hepatic stellate cells (HSCs) by previous studies. At present, the activation of HSC is an important cause of liver fibrosis, and the induction of HSC apoptosis plays an irreplaceable role in reversing liver fibrosis. Therefore, it is of great significance to explore mechanisms of action that can induce HSC apoptosis for the reversal of hepatic fibrosis and the clinical prevention and treatment of hepatic-fibrosis-related diseases such as hepatitis, cirrhosis, and liver cancer. In the current study, we demonstrated that tunicamycin (a novel ERS inducer) can induce the apoptosis of HSCs and increase the concentration of intracellular Ca^2+^ and the expression of ERS protein GRP78, apoptosis protein caspase-12, and Bax, while it can decrease the antiapoptosis protein expression of Bcl-2. Our findings indicate that tunicamycin can induce HSCs apoptosis through calpain-2/Ca^2+^-dependent ERS pathway.

## Introduction

Hepatic fibrosis is an essential intermediate process in the development of chronic liver disease to cirrhosis and even liver cancer (Tanwar et al., 2020). Activation of hepatic stellate cells (HSCs) is central to the development of hepatic fibrosis (Huang et al., 2020). When HSCs are activated, they transform into myofibroblasts and secrete a large number of fibers, and the degradation of fibers decreases relatively or absolutely, which results in excessive production of extracellular matrix and the increase in type I and type III collagen synthesized by cells (Jin et al., 2016; Matsuda and Seki, 2020). Hepatic fibrosis can be alleviated or reversed by inhibiting HSCs proliferation and inducting HSCs apoptosis (Bai et al., 2016; Xu T. et al., 2016).

It has been reported that endoplasmic reticulum stress (ERS)-mediated apoptosis plays an important role in the treatment of liver fibrosis, and calcium-dependent cysteine protease (calpain)/caspase-12 pathways are closely related to apoptosis (Huang et al., 2014). Studies have shown that regulating calpain-2 can affect the change in intracellular Ca^2+^ concentration (Nagy et al., 2016; Sasaki et al., 2016; Li et al., 2018). However, the mechanism of calpain-2/Ca^2+^-dependent ERS pathway in HSCs apoptosis has not been elucidated. In the study, ERS inducer tunicamycin (TM) was used to treat rat HSCs stimulated by transforming growth factor-β1 (TGF-β1) (the strongest profibrotic factor), and calpain-2 inhibitor N-acetyl-Leu-Leu-Norleucinal (ALLN) was used to inhibit calpain-2, then observed the related results. The aim is to explore the roles of preventing and treating hepatic fibrosis *via* calpain-2/Ca^2+^-dependent ERS pathway.

## Materials and Methods

### Cell Culture

The HSC line HSC-T6 isolated from carbon tetrachloride-stimulated rats was obtained by Professor Greenwell from the cell bank of George Washington University in the United States. The cells were cultured in Dulbecco’s modified Eagle’s medium (DMEM; # 2111072; Biological Industries Corporation, Kibbutz Beit Haemek, Israel) supplemented with 10% fetal bovine serum (FBS; # 04-001-1B; Biological Industries Corporation, Israel), 100 U/ml penicillin, and 100 μg/ml streptomycin in a humidified air at 37°C with 5% CO_2_ incubator. When the cells grow at a single layer of densification, they were dislodged by trypsin (# 25200056; Gibco Corporation, New York, United States) and seeded in culture bottles.

### Tunicamycin Concentration and Treatment Time Screening

The concentration and treatment time of TM (# 5045700001; Sigma Corporation, Missouri, United States) were screened by 3-(4,5-dimethylthiazol-2-yl)-2,5-diphenyltetrazolium bromide (MTT) (# D0801; Tokyo Chemical Industry, Tokyo, Japan) method. The cells were divided into four groups after synchronization and treated with 0, 1, 2, and 4 μg/ml TM for 24 h, respectively. Twenty microliters of MTT was added to the cells in the dark, and they were incubated at 37 C with 5% CO_2_ for 4 h. One hundred fifty microliters of dimethyl sulfoxide (DMSO) solution per well was added to the cells after discarding the culture medium. After the HSCs were oscillated at low speed for 10 min, the absorbance (OD) value was read and the proliferation rate (PR) (PR = T/C × 100%, T was the OD value of the treatment group, and C was the OD value of the 0 μg/ml TM group) was calculated after adjusting the wavelength of the plate analyzer to 490 nm. The cells were divided into three groups after synchronization and treated with 2 μg/ml TM for 12, 24, and 36 h, respectively. The OD value was detected by the same method as above.

### Cell Grouping and Treatment

The cells were cultured in serum-free DMEM medium for 24 h and then divided into the following groups: blank group, TGF-β1 group, TGF-β1 + ALLN + TM group (ALLN + TM group), TGF-β1 + TM group (TM group). The cells in the blank group were cultured in DMEM without FBS for 48 h and those in the other three groups were treated with 5 ng/ml TGF-β1 (# 100-B-010; R&D Systems, United States) for 24 h; then, the TGF-β1 group was cultured with blank medium for 24 h. The ALLN + TM group was pretreated with 25 μmol/l ALLN (# 110044-82-1; Selleck Corporation, Houston, TX, United States) for 30 min, and 2 μg/ml TM was added to the ALLN + TM group for 24 h; 2 μg/ml TM was added to the TM group and cultured for 24 h. All groups were incubated for 48 h.

### Observation of Hepatic Stellate Cells Ultrastructure by Transmission Electron Microscope

Cells were cultured conventionally, digested by trypsin, centrifuged, and washed twice with the precooled phosphate-buffered saline (PBS), fixed with glutaraldehyde at 4°C overnight, fixed with 1% citric acid for 2 h, dehydrated with alcohol and acetone, embedded with epoxy resin, sliced, and stained with uranyl acetate and lead citrate; then, the cells were observed by transmission electron microscopy (HITACHI Corporation, Osaka, Japan).

### Detection of Cell Cycle by Flow Cytometry

After HSCs were treated with corresponding reagents, the cells were digested with 2.5 g/L trypsin to be dispersive cells, then collected by centrifugation, and the supernatant was discarded. The cells were washed twice with precooled PBS, and 75% ethanol solution was added into the cell deposition at 4°C overnight. Cells were collected by centrifugation, washed twice with precooled PBS to resuspend the cells, then added 500 μl PBS, 50 μl propidium iodide (PI) (100 μg/ml; # P4170; Sigma Corporation, United States) and 2 μl RNase A. Finally, the cells were examined using flow cytometry (BD Corporation, New Jersey, United States).

### Cell Apoptosis Assay

Apoptosis of HSCs was observed by acridine orange/propidium iodide (AO/PI) staining. The cells were digested and counted on the counting plate. The cell concentration was adjusted to 2 × 10^4^/ml. The cells were cultured in an incubator at 37°C for 24 h and observed under the microscope. When the cells grew to 80% densities, they were cultured with blank medium for 24 h to synchronize. After the cells in different groups were treated, 10 μl of the prepared AO (100 μg/ml; # Sigma A6014; Sigma Corporation, United States) and PI solution (100 μg/ml; # P4170; Sigma Corporation, United States) was added to the cells in the dark condition. After shading reaction for 15 min at room temperature, the cells were observed and photographed under a fluorescence microscope.

### Ca^2+^ Fluorescence Intensity Assay

Ca^2+^ fluorescence intensity of HSCs was observed by laser scanning confocal microscopy (Olympus Corporation, Tokyo, Japan). The cells were digested, and their density was adjusted to 2 × 10^4^/ml, and the confocal dishes were used for subculture inoculation. After the cells were treated with the corresponding group, they were rinsed twice with 1 ml PBS, and then, 1 ml blank culture medium containing 1% Fluo-3AM (# F1241; Thermo Fisher Scientific, Waltham, MA, United States) was added to each dish, and the cells were cultured in an incubator at 37°C for 40 min in the dark. The cells were rinsed three times with PBS and further cultured in the incubator for 20 min after adding 1 ml complete medium. The excitation wavelength of the confocal microscope was set as 488 nm, and the emission wavelength was set as 530 nm. FV10-ASW 4.0 Viewer was used to process the image data. Six cells were selected from each image to calculate the fluorescence intensity.

### Detection of Calpain-2 and Caspase-12 Protein by Immunocytochemistry

The cells were digested, inoculated on coverslips and treated for a specified time, fixed with 4% paraformaldehyde solution, and operated according to the instructions of the immunocytochemical staining kit. The concentration of calpain-2 and caspase-12 primary antibody (anti-calpain-2 antibody: # A03492; Boster Biological Technology Co., Ltd., Wuhan, China) (anti-caspase-12 antibody: # bs-23014R; Bioss Biological Co. Ltd., Beijing, China) was 1:400. The positive result of protein expression was the deposition of dark brown particles in HSCs. The stained cells were photographed, and the optical density was detected and analyzed by Image-Pro Plus software.

### Western Blot Assay for GRP78, Bax, and Bcl-2

The cells of four groups were collected and rinsed with precooled PBS for three times. The culture dish was placed on ice and added with 200 μl cell lysis buffers [protease inhibitor: radioimmunoprecipitation assay (RIPA) = 1:250]. The protein samples were extracted and placed in 4°C refrigerators for 30 min, then centrifuged (12,000 rpm; 4°C) for 15 min. The supernatant was taken, and the volume was recorded. BCA kit (#70-PQ0011; MultiSciences Biotech, Co., Ltd., Hangzhou, China) was used for quantitative detection of protein concentration. LDS sample buffer (5×) and RIPA lysate were mixed in proportion, trimmed, and then added to the supernatant and boiled at 100 C for 5 min and stored at −20 C for use.

The expressions of GRP78, Bax, and Bcl-2 were detected by sodium dodecyl sulfate–polyacrylamide gel electrophoresis (SDS-PAGE). After blocking, the cells were transferred to polyvinylidene fluoride (PVDF) membrane using a wet transfer method. The primary antibodies, namely, anti-GRP78 antibody (1:200; # bsm-51623M; Bioss Biological Co. Ltd., Beijing, China), anti-Bax antibody (1:200; # BA0315-2; Boster Biological Technology Co., Ltd., Wuhan, China), and anti-Bcl-2 antibody (1:200; # A00040-2; Boster Biological Technology Co., Ltd., Wuhan, China) were incubated overnight at 4 C. Then, they were washed with Tris-buffered saline with Tween 20 (TBST) three times, and the secondary antibody, namely, horseradish peroxidase-conjugated antirabbit (1:5,000; # ZB2307, Zsbio Commerce Store, Beijing, China) was incubated at 37 C for 1 h and washed with TBST three times. Equivalent mixed ECL luminescence reagents A and B (# 32109; Thermo Fisher Scientific, United States) were used to detect the protein bands, and the gray values of protein bands were analyzed by an imaging analyzer (Bio-Rad Company, California, United States).

### Statistical Analysis

The SPSS 19.0 software was used for statistical analysis of the data. Results were described as the means ± SD; the comparison of multiple groups was performed by one-way analysis of variance. The comparison between groups was performed by least significant difference (LSD) *t*-test. In all cases, *p* < 0.05 was considered to be statistically significant.

## Results

### Tunicamycin Concentration and Treatment Time Screening by 3-(4,5-Dimethylthiazol-2-yl)-2,5-Diphenyltetrazolium Bromide Method

**FIGURE 1:**
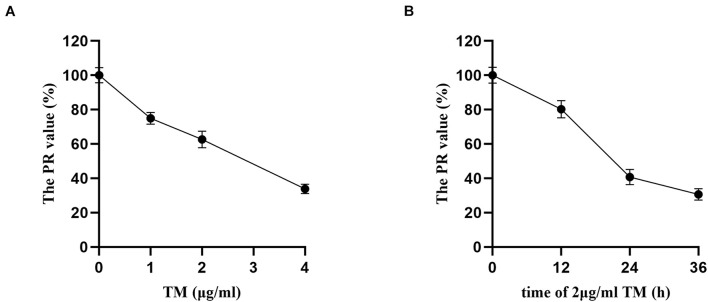
Tunicamycin concentration and treatment time screening by MTT. After the HSCs were treated by TM with different concentrations and treatment time, the OD value of each treatment was assayed, and the proliferation rate (PR) of HSCs was calculated. **(A)** HSCs were treated with various concentrations of TM for 24 h. **(B)** HSCs were incubated in the presence of 2 μg/ml TM for the indicated time periods.

Proliferation rate values of HSCs in the TM groups with different concentrations (0, 1, 2, and 4 μg/ml) were statistically significant (*p* < 0.05). With the increase in TM dose, the PR values decreased. There were significant differences in the PR value in others (74.95, 62.63, and 33.85%) compared with TM 0 μg/ml group (*p* < 0.05), and the PR value of 4 μg/ml group was only 33.85%. Therefore, the effective dose of TM was 2 μg/ml (Figure 1A).

Hepatic stellate cells were incubated with TM 2 μg/ml for 12, 24, and 36 h, and there were significant differences of PR values in the three groups (*p* < 0.05). PR value reached the maximum after 12 h (80.21%) and the minimum after 36 h (30.67%). The PR value of TM 2 μg/ml treated for 36 h was significantly lower than that of the 24 h group (40.79%) (*p* < 0.05). As the PR value of TM 2 μg/ml reduced significantly after 36 h of HSC treatment, 24 h was selected as the optimal treatment time of TM (Figure 1B).

### Morphological Changes in Hepatic Stellate Cells Were Observed by Transmission Electron Microscope

**FIGURE 2:**
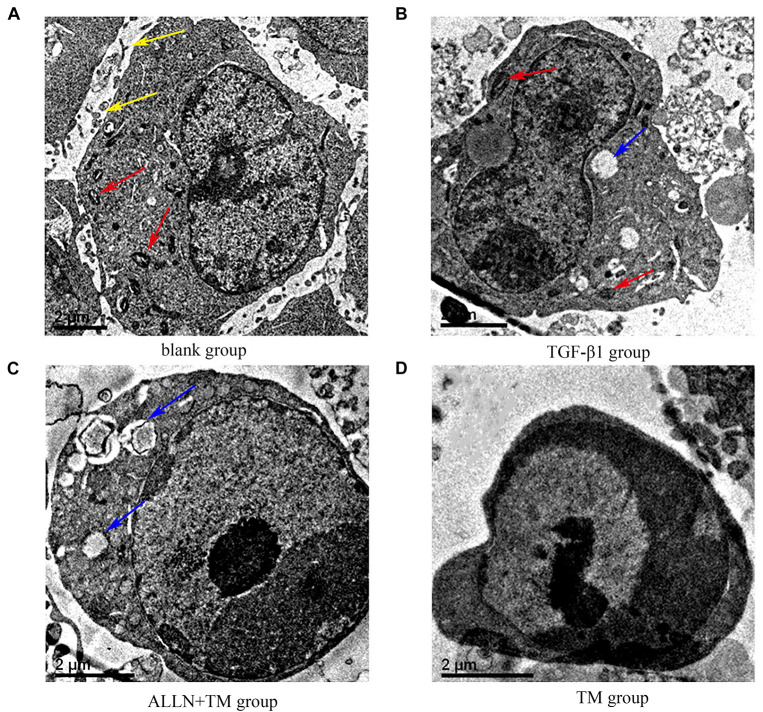
The ultrastructural changes of HSC in each group. **(A)** Control group was cultured in complete medium for 48 h. **(B)** TGF-β1 group was cultured with TGF-β1 blank medium for 48 h. **(C)** TGF-β1 + ALLN + TM group was cultured with the same concentration of TGF-β1 blank culture medium for 24 h, then pretreated with ALLN for 30 min and added TM for 24 h. **(D)** TGF-β1 + TM group was incubated with TGF-β1 blank medium for the same time, then added TM for 24 h; yellow arrows marked the villi, red arrows marked the mitochondria, and blue arrows marked the vacuoles. TM, tunicamycin; ALLN, N-acetyl-leu-leu-norleucinal; HSC, hepatic stellate cell; TGF-β1, transforming growth factor-β1.

The ultrastructural changes in cells were observed by transmission electron microscopy (Figure 2). In the blank group, HSCs had clear nucleoli, abundant surface villi, and even distribution of chromatin. Organelles with complete morphology and normal number, such as mitochondria, were seen in the cytoplasm (Figure 2A). HSCs in the TGF-β1 group had uniform and dense chromatin, clear nuclei, partial normal mitochondria, and partial vesicles (Figure 2B). However, in the ALLN + TM group, the microvilli were shed, the intercellular space became larger, vacuoles formed after organelle disintegration, and chromatin had slight marginal aggregation (Figure 2C). At the same time, in the TM group, the nuclei were seriously condensed and deformed, and the chromatin had obvious “crescent shape” changes (Figure 2D).

### Changes in Hepatic Stellate Cells Cycle in Each Group

**FIGURE 3:**
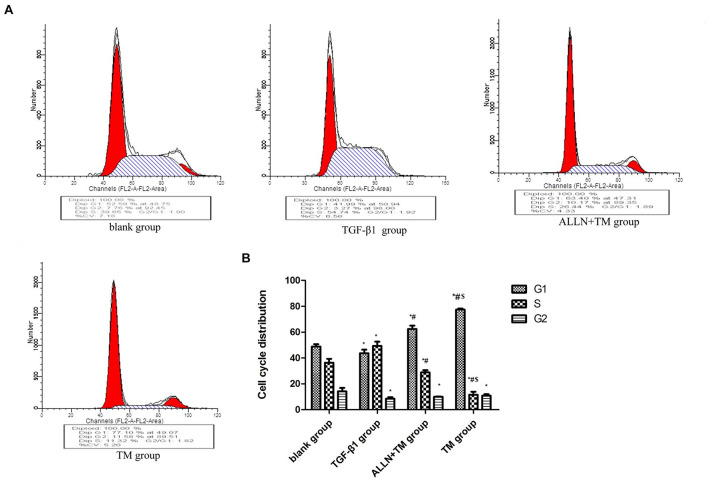
The cell cycle changes in HSC in each group. After treated with TGF-β1, ALLN, and TM, HSC cell cycle of each group was determined by flow cytometric analysis. **(A)** Figures represent the distribution of different cell cycle phase. **(B)** Columns represent the percentages of the corresponding cell cycle phase. (*p < 0.05 compared with the blank group; ^#^*p* < 0.05 compared with the TGF-β1 group; ^$^*p* < 0.05 compared with the ALLN + TM group).

Changes in HSCs cycle were analyzed by flow cytometry (Figure 3A). The differences in cell cycle in the treatment groups had statistical significance (*p* < 0.05). Compared with the blank group, the proportion of cells in G1 phase decreased (*p* < 0.05), which increased in S phase (*p* < 0.05) and decreased in G2 phase (*p* < 0.05) in the TGF-β1 group. When compared with TGF-β1 group, the proportions of cells in G1 phase were significantly higher than that in the ALLN + TM group and the TM group (*p* < 0.05), while they were significantly lower in S phase (*p* < 0.05) and did not change significantly in G2 phase (*p* > 0.05). The proportion of G1 phase cells in the TM group was significantly higher than that in ALLN + TM group (*p* < 0.05), while the proportion of S phase was significantly lower (*p* < 0.05), and the change in G2 phase was not obvious (*p* > 0.05), as shown in Figure 3B.

### Effects of Different Treatments on Apoptosis of Hepatic Stellate Cells

**FIGURE 4:**
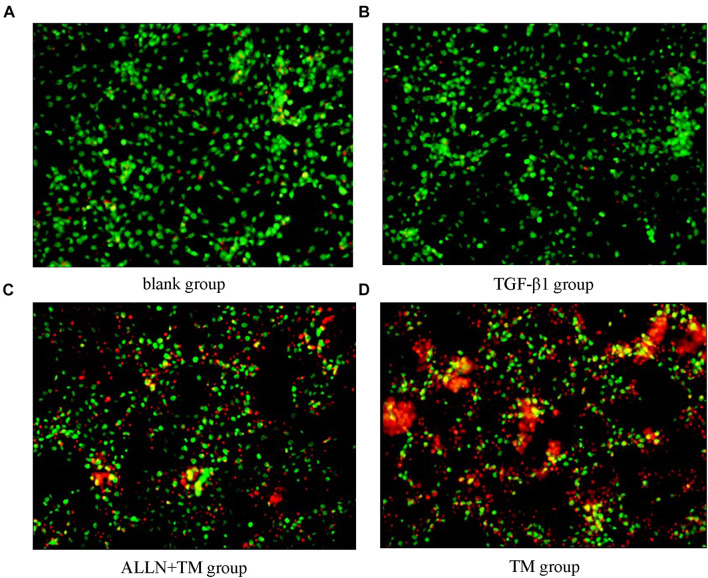
Cell apoptosis was observed by AO/PI staining. Cell apoptosis was detected by staining with acridine orange/propidium iodide (AO/PI) after treatment with TGF-β1, ALLN, and TM in HSC. **(A)** Blank group. **(B)** TGF-β1 group. **(C)** ALLN+TM group. **(D)** TM group. Green fluorescence represented survival, and red or orange represented apoptosis.

The cells were double stained by AO/PI and observed under a fluorescence microscope, in which green fluorescence marked surviving cells and red/orange marked apoptotic cells. Most of the cells in the blank group showed green fluorescence, meaning that they were growing well (Figure 4A). HSCs in the TGF-β1 group was alive and grew vigorously (Figure 4B). There were some small and medium-sized cells in the ALLN + TM group showing red/orange fluorescence, and green fluorescence still accounted for the majority (Figure 4C). Most of the cells in the TM group showed red/orange fluorescence, and the green fluorescence weakened (Figure 4D).

### Changes in Intracellular Ca^2+^ Concentration

**FIGURE 5:**
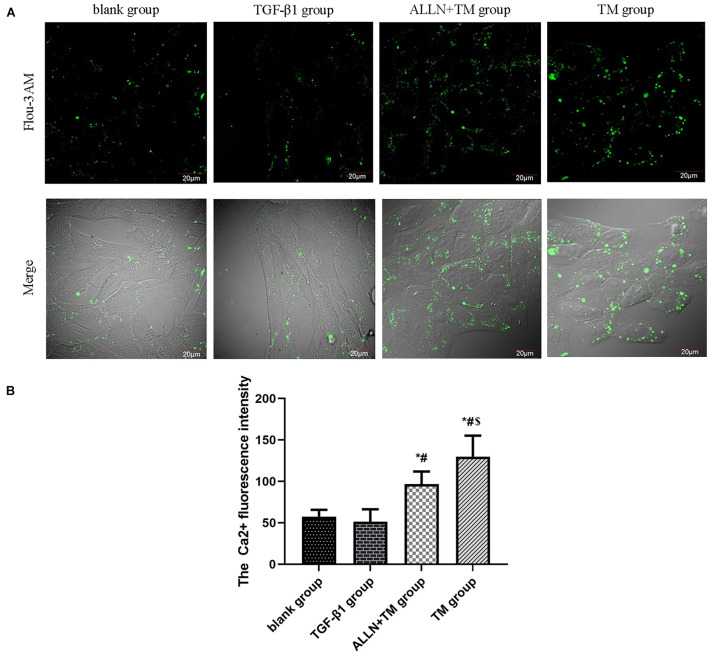
The changes in Ca^2+^ fluorescence intensity in HSC. The changes in Ca^2+^ concentration in each group were detected by a laser scanning confocal microscope. **(A)** Green fluorescence represents the Ca^2+^ fluorescence intensity in HSC. **(B)** Columns represent Ca^2+^ fluorescence intensity in HSC (*p < 0.05 compared with the blank group; ^#^*p* < 0.05 compared with the TGF-β1 group; ^$^*p* < 0.05 compared with the ALLN + TM group).

Laser confocal microscopy results showed that Ca^2+^ fluorescence intensity of HSCs was slight in the blank and TGF-β1 groups, which was enhanced and strongest in the ALLN + TM and TM groups (Figure 5A). There was no significant difference in Ca^2+^ concentration between the blank group and TGF-β1 group (*p* > 0.05). Ca^2+^ concentration in the ALLN + TM and TM groups was significantly higher than that in the TGF-β1 group (*p* < 0.05), while in the ALLN + TM group, it was significantly lower than that in the TM group (*p* < 0.05) (Figure 5B).

### The Expression of Calpain-2 and Caspase-12 Protein

**FIGURE 6:**
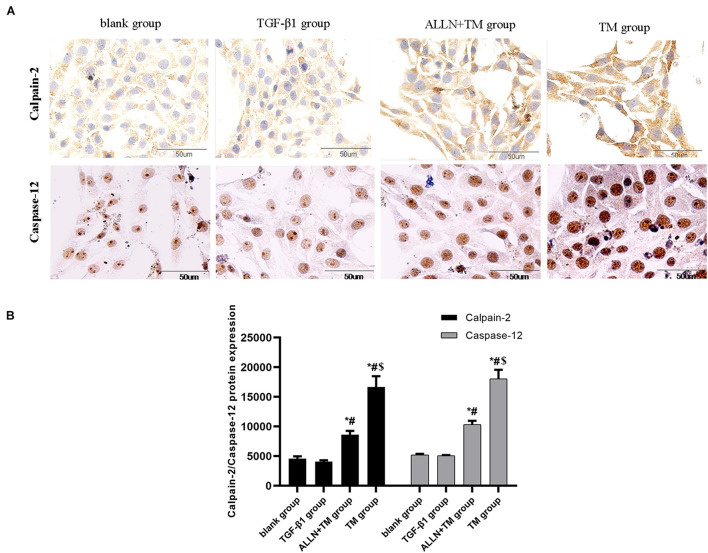
The protein expression level of calpain-2 and caspase-12 in HSC in different groups. Expressions of calpain-2 and caspase-12 protein were detected by immunocytochemistry. **(A)** Brown part in the figures represent the protein expression in HSC. **(B)** Columns represent protein expression level of calpain-2 and caspase-12 in HSC (*p < 0.05 compared with the blank group; ^#^*p* < 0.05 compared with the TGF-β1 group; ^$^*p* < 0.05 compared with the ALLN + TM group).

Immunocytochemistry results showed that the calpain-2 and caspase-12 proteins in HSCs were expressed (Figure 6A). Immunocytochemistry results of calpain-2 (cytoplasm) in the blank and TGF-β1 groups showed that it was weak and light brown, while in the ALLN + TM and TM groups, it was darker and showed heavy brown. The expression of calpain-2 in the ALLN + TM and TM groups was higher than that in the TGF-β1 group (*p* < 0.05), in which it was significantly lower in the ALLN + TM group than that in the TM group (*p* < 0.05) (Figure 6B).

The staining of caspase-12 protein was light brown in the blank and TGF-β1 groups, while in the ALLN + TM and TM groups, it was dark brown (Figure 6A). There was no significant difference in the expression of caspase-12 between blank group and TGF-β1 group (*p* > 0.05). Compared with the TGF-β1 group, the expression of caspase-12 protein in the ALLN + TM and TM groups were significantly increased (*p* < 0.05). The expression of caspase-12 protein in the TM group was significantly higher than that in the ALLN + TM group (*p* < 0.05) (Figure 6B).

### The Protein Expressions of GRP78, Bax, and Bcl-2

**FIGURE 7:**
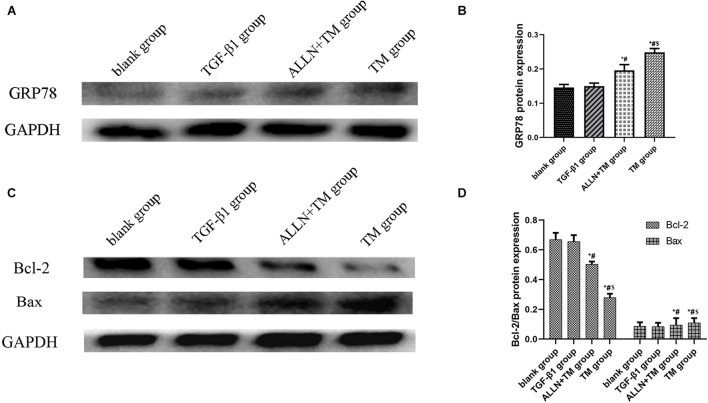
The protein expression level of GRP78, Bax, and Bcl-2 in different groups. The protein expression level of GRP78, Bax, and Bcl-2 were detected by western blot. GAPDH was used as a loading control. **(A,C)** The gel electrophoresis figure of GRP78, Bax, and Bcl-2 of different groups in HSC. **(B,D)** Related proteins gray value analysis (*p < 0.05 compared with the blank group; ^#^*p* < 0.05 compared with the TGF-β1 group; ^$^*p* < 0.05 compared with the ALLN + TM group).

The gel electrophoresis patterns of the three proteins are shown in Figures 7A,C. The expression of GRP78 had no significant difference between blank group and TGF-β1 group (*p* > 0.05), while in the ALLN + TM and TM groups, it was significantly higher than that in TGF-β1 group (*p* < 0.05). The expression of GRP78 in the TM group was significantly higher than that in the ALLN + TM group (*p* < 0.05) (Figure 7B). Compared with the blank group, the protein expression of Bax and Bcl-2 had no significant difference in the TGF-β1 group (*p* > 0.05). The expressions of Bax in the ALLN + TM and TM groups were significantly higher than that in the TGF-β1 group (*p* < 0.05), while the expression of Bcl-2 in the ALLN + TM and TM groups was lower than that in the TGF-β1 group (*p* < 0.05). The expression of Bax in the TM group was significantly higher than that in the ALLN + TM group (*p* < 0.05), while Bcl-2 in the TM group was significantly lower than that in the ALLN + TM group (*p* < 0.05) (Figure 7D).

## Discussion

Transforming growth factor-β1 is the strongest profibrotic factor (Blomgren et al., 2001; Dilshara et al., 2017) and significantly enhances the proliferative activity of HSCs (Wang et al., 2010). In this study, the proportion of HSC in S phase was significantly increased after TGF-β1 treatment. After TM treatment, the proportion of cells in G1 phase was significantly increased, while the proportion of cells in S phase was significantly decreased. Studies have shown that TM inhibits the cell cycle by blocking the G1 phase and reducing the proportion of S phase (Lu et al., 2016; Zheng et al., 2018). The inhibition of TM on DNA synthesis may be the cause of TM inhibiting the proliferation and promoti the apoptosis. Compared with TM treatment, G1 phase was significantly shortened, and S phase was significantly prolonged after ALLN intervention. These indicated that ALLN pretreatment adjusted the cell cycle <, and the result was consistent with relevant reports (Perrin et al., 2003).

Endoplasmic reticulum (ER) is the major reservoir of intracellular Ca^2+^. In the early stage of apoptosis, the outflow of Ca^2+^ in ER leads to the overload of Ca^2+^ in the cytoplasm and apoptosis (Kitano and Bloomston, 2016). It has been proven that TM increased Ca^2+^ concentration in oral cancer cells (Yu et al., 2016). The intracellular Ca^2+^ concentration of myocardial cells decreased after ALLN pre-perfusion for 15 min in isolated heart (Yuan et al., 2016). In this study, after HSCs were treated with TGF-β1, the fluorescence intensity in the cytoplasm was weak, Ca^2+^ concentration got reduced, When HSCs were treated by TM, the intracellular Ca^2+^ concentration increased significantly, while ALLN decreased the Ca^2+^ concentration by blocking calpain-2.

It has been reported that Ca^2+^ overload leads to neuronal apoptosis through calpain-2, when cerebral ischemia and hypoxia occur (Wang et al., 2018b). The expression of calpain-2 was significantly upregulated when TM induced ERS in human breast cancer cells (Han et al., 2015). Studies have shown that blocking the ERS pathway by ALLN pretreatment can significantly prolong the survival rate of liver cancer cells (Wang et al., 2018a). In this study, immunocytochemical staining results showed that calpain-2 was expressed in the cytoplasm of HSC. After TM treatment alone, the expression of calpain-2 in HSC increased, and the apoptosis of HSC was obvious. After ALLN pretreatment, the expression of calpain-2 decreased significantly, and the degree of HSC apoptosis decreased relatively. It is suggested that calpain-2 is involved in the apoptosis of HSC.

Endoplasmic reticulum stress pathway is a new apoptosis pathway discovered in recent years, which performs apoptosis by activating caspase-12 (Zhang et al., 2016). External factors act on ER to disrupt ER homeostasis, then unfolded protein reaction (UPR) occurs in ER to reconstruct the homeostasis, and GRP78 increases rapidly (Lombardi and Tomer, 2017). GRP78 is a specific signal molecule for ERS. In this study, GRP78 expression was significantly upregulated by TM, which was consistent with the research results of Brenner et al. (2013). Caspase-12 is a key molecule in cell apoptosis. When ERS induces the apoptosis, the expression of apoptosis protein caspase-12 increases with the increase in apoptosis (Wang et al., 2014). Calpain-2 acts on Bax to produce active fragments, which further regulates Bcl-2 and mediates apoptosis through caspase-12 (Chang et al., 2015; Xu P. et al., 2016). Bcl-2 is an inhibitor of apoptosis, and Bax is a promoter of apoptosis. The increase in Bcl-2 plays an important role in the development of heart failure and chronic liver disease (Tao et al., 2016; Mihailidou et al., 2017). We found that caspase-12 was expressed in stellate nuclei, and the nuclear staining deepened and showed dark brown with the increase in the apoptosis. The results in the study suggested that TM promoted cell apoptosis, and ALLN alleviated the degree of TM-induced apoptosis by blocking calpain-2. Transmission electron microscopy results also confirmed that ALLN disintegrated organelles into vacuoles, and chromatin had a slight edge deviation. It indicated that the degree of apoptosis was relatively mild, and ALLN inhibited TM-induced apoptosis. ALLN can decrease the activity of calpain-2 and affect the apoptosis.

In summary, the mechanism of TM effect on HSC apoptosis through calpain-2/Ca^2+^-induced ERS pathway was explored through *in vitro* cell experiments in the current study, which further needs to be verified by animal experiments *in vivo*. This study clarified the new mechanism of inducing HSCs apoptosis and provides a new target for clinical development of anti-fibrosis drugs.

## Data Availability Statement

The original contributions presented in the study are included in the article/supplementary material, further inquiries can be directed to the corresponding author.

## Author Contributions

YX and FF created the study concept and designed the experiments. HL performed the experiments and wrote the manuscript. MW and LD analyzed the data. All authors read and approved the final manuscript.

## Conflict of Interest

The authors declare that the research was conducted in the absence of any commercial or financial relationships that could be construed as a potential conflict of interest.

## Publisher’s Note

All claims expressed in this article are solely those of the authors and do not necessarily represent those of their affiliated organizations, or those of the publisher, the editors and the reviewers. Any product that may be evaluated in this article, or claim that may be made by its manufacturer, is not guaranteed or endorsed by the publisher.
